# Graduate and Health Professional Student Knowledge, Attitudes, Beliefs, and Behavior Related to Human Papillomavirus and Human Papillomavirus Vaccination: A Scoping Review of the Literature

**DOI:** 10.3390/vaccines12050507

**Published:** 2024-05-07

**Authors:** Joshua Gautreaux, Eric Pittman, Kennedy LaPorte, Jiaxin Yang, Marie Barnard

**Affiliations:** 1 Department of Health, Exercise Science and Recreation Management, School of Applied Sciences, University of Mississippi, University, MS 38677, USA; jmgautre@go.olemiss.edu; 2 Department of Pharmacy Administration, School of Pharmacy, University of Mississippi, University, MS 38677, USA; epittman@olemiss.edu (E.P.); kclaport@go.olemiss.edu (K.L.); jyang7@go.olemiss.edu (J.Y.)

**Keywords:** human papillomavirus (HPV), vaccine, health education, graduate students, college, scoping review

## Abstract

Human papillomavirus (HPV) is a common sexually transmitted infection. Despite a safe and effective vaccine, uptake continues to be suboptimal. Recently, focus has moved to college campuses in an effort to increase vaccination rates. Little is known about the extent of efforts to reach graduate students on college campuses in the United States and the vaccination rates within this subpopulation. This scoping review assessed the literature on knowledge, attitudes, beliefs, and behaviors about HPV and HPV vaccination among graduate and post-baccalaureate professional students in the United States. This review also aims to identify areas for further research to improve institutions’ abilities to create health programming to increase HPV awareness and HPV vaccination coverage on their campuses. Publications focusing on knowledge, attitudes, beliefs, and behaviors about HPV and HPV vaccination in post-baccalaureate students were included. The systematic review of PubMed, CINAHL, and Embase identified 2562 articles, and 56 articles met all inclusion criteria and were included in this scoping review. A majority of the reviewed studies investigated some combination of knowledge, attitudes, behaviors, and beliefs about HPV and the HPV vaccine in students in professional programs such as medicine. Study design approaches were primarily cross-sectional, utilizing web-based survey distribution methods. HPV vaccination status and HPV screening behaviors were primarily measured through participant self-report. There is limited research investigating post-baccalaureate student knowledge, attitudes, beliefs, and behaviors about HPV and HPV vaccination. There is a need for researchers to further investigate the needs of graduate students to create informative and effective HPV programming.

## 1. Introduction

Human papillomavirus (HPV) is a common and highly transmissible sexually transmitted infection that affects approximately 13 million Americans per year [1]. HPV infection has been implicated as a primary cause of anal, cervical, head and neck, vulvar, penile, and vaginal cancers [2]. While HPV-related cervical cancer rates in women are on the decline due to HPV vaccination, there has been an increase in head, neck, and rectal cancer rates in men [3]. In total, about 37,000 new cancer cases are caused by HPV each year [4]. Despite the existence of a safe and effective vaccination that protects against multiple highly problematic strains of HPV, vaccination uptake rates in the United States continue to be suboptimal, including in adolescents [5].

A recent study investigating the behavioral trends of parents of HPV vaccination-eligible adolescents highlighted a general mistrust and concerns about safety as primary reasons for the delay or outright refusal of the vaccine [6]. Negative parental beliefs about the HPV vaccination included a concern for harmful side-effects, a lack of knowledge of the components of the vaccine, and a disbelief that the vaccine is useful as a cancer prevention tool [6,7]. Less commonly reported reasons for vaccination hesitancy and refusal among parents included a lack of recommendation from primary care providers, the potential for sexual promiscuity, and concerns about the cost of multiple vaccinations to complete the series [6]. The lack of adherence to vaccination recommendations among concerned parents could leave many young individuals unprotected and at risk of developing an HPV-related cancer later in life.

Given the serious implications of HPV infection and the low adolescent vaccination rate (58.5%), offering catch-up vaccinations in college has become a significant area of interest [5]. College is often the first time young adults are acting independently of their parents as it relates to decisions on their personal health and wellbeing. In order to understand the extent of HPV vaccination coverage, many studies on college campuses have investigated vaccination rates and perceptions of the HPV vaccine among undergraduate student populations [8,9,10]. While the awareness of HPV and the HPV vaccine in undergraduate college students was found to be high, when asked to verify facts about the vaccine, many students were found to have significant gaps in their knowledge about the safety and effectiveness of the vaccine, which could influence vaccination-seeking behaviors [10]. Students were also found to have a lack of understanding of their susceptibility and risk of contracting HPV, as well as the potential long-term consequences of infection [10]. Overall, college-aged females were more likely to be vaccinated than their male peers, though males have seen increases in vaccination rates in recent years [9]. However, college students, male and female, who were older in age were also less likely to have been vaccinated against HPV [9].

In 2019, the Advisory Committee on Immunization Practices (ACIP) recommended that individuals through the age of 26 were eligible to receive HPV catch-up vaccinations [11]. The ACIP concurrently released guidance that stated that individuals in the 27–45 age range should discuss with their primary care provider their eligibility to receive the HPV vaccination. The latest ACIP recommendations extended the age range of individual eligibility to receive the HPV vaccine, thus creating an opportunity to reach graduate and professional students with informative and effective programming to increase HPV vaccination coverage. Understanding the landscape of graduate and professional student knowledge, attitudes, beliefs, and behaviors about HPV and HPV vaccination is an important step in achieving optimal vaccination rates in this subpopulation.

## 2. Methods

This review utilized a framework originally proposed by Arksey and O’Malley with adaptations made by Levac, Colqohoun, and O’Brien as well as the Joanna Briggs Institute (JBI) [12,13,14]. Guidance from the Preferred Reporting Items for Systematic Reviews and Meta-Analyses for Scoping Reviews was used to develop the protocol for the review, which was deposited in the online open access repository Egrove [15,16].

## 3. Objective

The purpose of this scoping review is to explore, describe, and assess the extent of the literature on the current state of knowledge, attitudes, beliefs, and behaviors about HPV and HPV vaccination among graduate and post-baccalaureate professional students in the United States.

## 4. Search Strategy

PubMed, Embase, and CINAHL were searched to identify studies to include in this review. The search terms and strategy were drafted and finalized during multiple team discussions (see Appendix A for the search strings for each database).

### 4.1. Inclusion and Exclusion Criteria

In order to be included in the scoping review, publications had to have a focus on assessing or measuring knowledge, attitudes, behaviors, and beliefs about HPV and the HPV vaccination in post-baccalaureate college students in the United States. Studies also had to be available in English. Studies were not included if they did not include measurements or assessments on any of the aforementioned variables. Studies that did not include graduate or post-baccalaureate students were excluded. Given the lack of HPV-related studies specifically targeting post-baccalaureate students, studies that met the inclusion criteria frequently included undergraduate students.

### 4.2. Study Selection

Search results from each database were imported into the reference manager Zotero and duplicates were removed. Title and abstract reviews were then completed using independent reviewers working in pairs. Comparisons for congruence among raters was conducted with any discrepancies resolved by a third reviewer. Articles deemed eligible were then moved to a full-text review. Four reviewers independently screened articles to determine eligibility for inclusion. Any disagreements in this stage of the review were resolved by team meetings and discussion. The reason for exclusion for any article in this stage was recorded. A data extraction form was created in Qualtrics. This data extraction form was piloted and refined by the reviewers as a team. The data abstracted included study aims; study design; graduate student sample size; characteristics of the study population; study location; HPV vaccination status; HPV screening behaviors; knowledge, attitudes, behaviors, beliefs about HPV and the HPV vaccine; and the funding source. Two reviewers independently extracted data from each article. The concordance of the abstracted data was reviewed and adjudicated by a third reviewer. Data from these extractions were then organized into tables to characterize the studies and describe the variables of interest that were included in the studies reviewed.

## 5. Results

### 5.1. Selected Articles for Review

**Figure 1:**
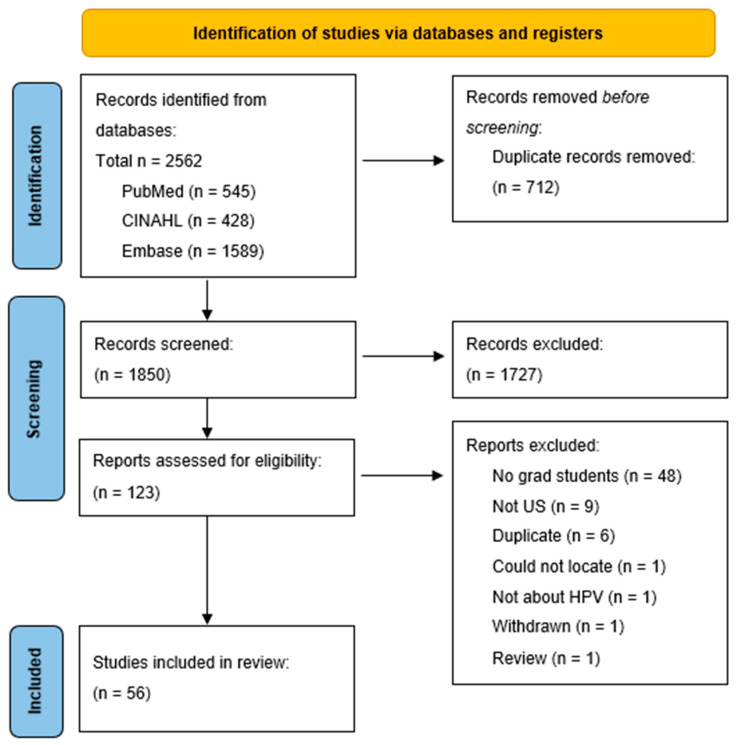
Preferred Reporting Items for Systematic Reviews and Meta Analyses (PRISMA) flowchart of article extraction from literature review [15].

A total of 2562 articles were identified from database searches of PubMed (545), CINAHL (428), and Embase (1589). Of the 2562 articles identified, 712 duplicates were removed. A total of 1850 articles moved forward in the screening process. Upon completion of title and abstract screening, 1727 articles were removed, leaving 123 articles to be assessed for eligibility by full-text review. A total of 67 articles were excluded in this stage and a final total of 56 articles were identified for inclusion in the review as seen in Figure 1.

### 5.2. Descriptive Analysis of Articles

The 56 articles included in the scoping review consisted of studies that were primarily conducted at medical schools and university campuses. The primary modality of included studies were surveys, usually performed online. A majority of the studies included in the review utilized a cross-sectional approach (73%, 41/56). Intervention-based approaches were utilized in 23% of the studies (13/56). Moreno et al. [17] utilized a qualitative interview approach and Frank et al. [18] conducted a longitudinal study (see Table 1).

While all studies included in the final review must have studied post-baccalaureate participants, they often included various student populations including undergraduate students, graduate students, medical students, dental students, dental hygienists, nurse practitioners, professional students, health students, and medical and dental graduates in residency. Most of the studies focused on medical students, with 34% (19/56) of the reviewed articles including only medical students. Articles that included studies strictly on graduate and undergraduate students made up 25% (14/56) of the final review. None of the reviewed articles consisted of studies in which the focus was on a population of non-health-sciences graduate students.

Of the studies included in the review, 64% (36/56) included disaggregated outcome data based on the student population type. In 36% (20/56), outcome data were not disaggregated; therefore, there was no way to determine graduate student knowledge, attitudes, behaviors, or beliefs from the information provided. The total number of post-baccalaureate students included in all the articles included in this review was 36,719. Disaggregated outcome data for students that were classified as traditional graduate students (non-health-sciences graduate students) were available for less than 5% of the total sample (1740 /36,563).

### 5.3. Knowledge, Attitudes, Beliefs, and Behaviors Related to HPV and HPV Vaccination

Studies that investigated knowledge about HPV accounted for 79% (44/56) of the articles in the final review. Knowledge about the HPV vaccine was assessed in 75% (42/56) of the articles. Awareness about HPV was assessed in 21% (12/56) and awareness about the HPV vaccine, including safety and efficacy, was assessed in 25% of the articles (14/56). Attitudes and beliefs about HPV were assessed in 50% (28/56) and attitudes and beliefs about the HPV vaccine were assessed in 68% (38/56) of articles. HPV vaccination behavior measured as self-reported HPV vaccination status was assessed in 61% (34/56) of articles. Only two of the studies reviewed participant health records to verify vaccination status. HPV-related screening behaviors such as pap smears were assessed in 7% (4/56) of articles (see Table 2).

## 6. Discussion

The aim of this scoping review was to explore, describe, and assess the literature on the current state of knowledge, attitudes, behaviors, and beliefs about HPV and the HPV vaccine among graduate and post-baccalaureate student populations. Uptake of the HPV vaccine in adolescents in the US is suboptimal due to parental concern and hesitancy, leading to significant cancer prevention gaps. Colleges and universities equipped with campus clinics and health and wellness programs have a unique opportunity to provide education and vaccination opportunities for students to increase vaccination rates. While there have been documented success in increasing knowledge, attitudes, behaviors, and beliefs in undergraduate students through educational campaigns, the awareness and educational needs of graduate students may differ from undergraduate students.

Investigating the specific needs of graduate students is important to inform the development of appropriate interventions for this at-risk population. This scoping review identified 56 eligible studies that included outcome measures on knowledge, attitudes, behaviors, and beliefs around HPV and the HPV vaccine in graduate and post-baccalaureate students. An overwhelming majority of the studies that investigated knowledge, attitudes, behaviors, and beliefs about HPV and the HPV vaccination utilized cross-sectional study designs with self-reported survey data. Many of the identified studies included various student populations including undergraduate, nursing, dental hygiene, and non-degree-seeking students. While these studies provided a broad understanding of measures such as an awareness of HPV or vaccine status on their campuses, a vital understanding of the nuances of the unique needs of graduate student populations was lacking.

Further complicating the understanding of graduate and post-baccalaureate students is the fact that many of the studies did not report disaggregated result data. Those studies that did report disaggregated data for graduate students generally had small sample sizes. The majority of students included in the studies were those in graduate health sciences programs (e.g., medicine, dentistry). While these students are important as they will be providing guidance related to HPV and the HPV vaccine to patients in the future, they likely already had a higher level of knowledge and awareness related to HPV and HPV vaccination, making it less valuable to extrapolate from the findings in those studies to students in other types of graduate programs. Future studies could examine potential differences in knowledge, attitudes, behaviors, and beliefs between health professional post-baccalaureate students and traditional graduate students. Future investigations may also be warranted to compare HPV knowledge, attitudes, behaviors, and beliefs between post-baccalaureate students and their work-force peers.

There are several limitations of this scoping review. The studies included in this scoping review were published in peer-reviewed journals found in the PubMed, Embase, and CINAHL databases. It is possible that relevant studies were published in journals not indexed in these databases. Additionally, awareness campaigns and vaccination interventions that may have been completed on college campuses with a focus on graduate or post-baccalaureate students but were not published could not be included. Another potential limitation is that many of the studies utilized survey measurement tools that depended on self-reported data. With a lack of access or investigation of vaccination records, it is impossible to verify the actual vaccination status of surveyed participants in the majority of studies. This review was limited to institutions of higher learning within the United States; therefore, broad generalizations about HPV and HPV vaccination knowledge, attitudes, behaviors, and beliefs cannot be inferred at the international level. Given the inherent differences that exist in HPV vaccination policy and procedures, a country-by-country evaluation of the literature is recommended.

Creating inclusive and informative campaigns on the various types of college campuses that target graduate and post-baccalaureate students is necessary to increase awareness of HPV and HPV vaccination and to increase HPV vaccine uptake. Currently, very little literature exists that explores knowledge, attitudes, behaviors, and beliefs around HPV and vaccination status in these groups as only 5% of the sample size in this review included graduate students. This scoping review highlights a gap in the literature and an opportunity for research focused on a specific population that is eligible for vaccination. Further investigation is necessary to understand the knowledge, attitudes, beliefs, and behaviors related to HPV and the HPV vaccine in graduate students in order to develop effective campaigns and intervention methods to increase uptake of this cancer-preventive vaccine.

## Figures and Tables

**Table 1:** Study design and sample characteristics.

Study	Study Design	Sample Description	Graduate Student Sample Size	Outcome Data Reported in a Disaggregated Manner by Student Group (Y/N)
Afonso et al. 2017 [19]	Cross-Sectional Study	Medical Students	390	Y
Albright et al. 2019 [20]	Cross-Sectional Study	Graduate and Undergraduate Students	16,370	N
Alsulami et al. 2023 [21]	Cross-Sectional Study	Graduate and Undergraduate Students	163	N
Barrera et al. 2021 [22]	Cross-Sectional Study	Medical, Graduate, and Undergraduate Students	65	Y
Bennett et al. 2015 [23]	Intervention Study	Graduate and Undergraduate Students	182	N
Berenson et al. 2021 [24]	Intervention Study	Medical Students and Graduate Students	512	Y
Berenson et al. 2017 [25]	Cross-Sectional Study	Medical Students	231	Y
Berenson et al. 2020 [26]	Intervention Study	Medical Students	256	Y
Blankenship et al. 2015 [27]	Cross-Sectional Study	Medical Students	308	Y
Buchanan et al. 2015 [28]	Cross-Sectional Study	Medical Students	345	Y
Bunting et al. 2023 [29]	Cross-Sectional Study	Medical Students	718	Y
Daniel et al. 2021 [30]	Cross-Sectional Study	Medical Students	127	Y
D’Errico et al. 2020—JAANP [31]	Cross-Sectional Study	Graduate and Undergraduate Students	194	N
D’Errico et al. 2020 Journal of NP [32]	Cross-Sectional Study	Graduate and Undergraduate Students	193	N
Du et al. 2022 [33]	Cross-Sectional Study	Medical Students	247	Y
Evans et al. 2020 [34]	Intervention Study	Medical, Graduate, Undergraduate, and Health Professional Students	230	Y
Franca et al. 2023 [35]	Cross-Sectional Study	Graduate and Undergraduate Students	241	Y
Frank et al. 2008 [18]	Longitudinal Study	Medical Students	2316	Y
Gerend and Magloire 2008 [36]	Cross-Sectional Study	Graduate and Undergraduate Students	14	N
Goldfarb et al. 2022 [37]	Cross-Sectional Study	Graduate and Undergraduate Students	14	N
Jacobson et al. 2020 [38]	Intervention Study	Medical Students	228	Y
Kepka et al. 2019 [39]	Cross-Sectional Study	Dental Students	233	N
Kim et al. 2019 [40]	Cross-Sectional Study	Graduate and Undergraduate Students	24	N
Kubli et al. 2017 [41]	Intervention Study	Graduate Professional Students	85	Y
Laitman et al. 2018 [42]	Cross-Sectional Study	Medical Students	617	Y
Laitman and Genden 2018 [43]	Cross-Sectional Study	Medical Students	1300	N
Laitman et al. 2020 [44]	Cross-Sectional Study	Medical Students	3141	Y
Levy et al. 2021 [45]	Cross-Sectional Study	Medical and Nursing Students	234	Y
Maginot et al. 2022 [46]	Cross-Sectional Study	Dental students and Dental Residents	293	Y
McCready et al. 2015 [47]	Cross-Sectional Study	Medical Students	85	Y
Moreno et al. 2017 [17]	Qualitative study	Graduate Students	7	N
Natipagon-Shah et al. 2021 [48]	Cross-Sectional Study	Graduate and Undergraduate Students	28	N
Navalpakam et al. 2016 [49]	Cross-Sectional Study	Graduate and Undergraduate Students	38	N
Nesser and Ayodele 2023 [50]	Cross-Sectional Study	Graduate and Undergraduate Students	755	N
Nkwonta et al. 2022 [51]	Cross-Sectional Study	Graduate and Undergraduate Students	42	N
Patel et al. 2012 [52]	Intervention Study	Graduate and Undergraduate Students	70	Y
Patel et al. 2013 [53]	Cross-Sectional Study	Graduate, Undergraduate, and Professional Students	1185	N
Richman et al. 2016 [54]	Intervention Study	Graduate and Undergraduate Students	33	Y
Richman et al. 2022 [55]	Intervention Study	Medical Students	103	Y
Rohde et al. 2018 [56]	Cross-Sectional Study	Medical and Undergraduate Students	248	Y
Ruebsamen et al. 2018 [57]	Cross-Sectional Study	Medical and Graduate Students	265	N
Rutkoski et al. 2020 [58]	Cross-Sectional Study	Dental Students and Dental Hygiene	297	Y
Schnaith et al. 2018 [59]	Intervention Study	Medical Students	132	Y
Shukla et al. 2022 [60]	Intervention Study	Dental and Undergraduate Students	125	N
Suryadevara et al. 2016 [61]	Cross-Sectional Study	Graduate and Undergraduate Students	222	Y
Sutton et al. 2021 [62]	Intervention Study	Medical Students	139	Y
Thompson et al. 2017 [63]	Cross-Sectional Study	Graduate and Undergraduate Students	836	Y
Torres et al. 2022 [64]	Cross-Sectional Study	Dental Students	109	Y
Tsau et al. 2011 [65]	Cross-Sectional Study	Medical, Graduate, and Health Professional Students	1119	N
Tung et al. 2019 [66]	Cross-Sectional Study	Graduate and Undergraduate Students	156	Y
Tung et al. 2021 [67]	Cross-Sectional Study	Graduate and Undergraduate Students	156	Y
Walker et al. 2018 [68]	Cross-Sectional Study	Dental, Nursing Students, Dental Hygiene, Nurse Practitioner	76	N
Wiley et al. 2018 [69]	Intervention Study	Medical Students	61	Y
Wiley et al. 2019 [70]	Cross-Sectional Study	Medical Students	895	Y
Wright et al. 2021 [71]	Cross-Sectional Study	Dental Students	173	Y
Yacobi et al. 1999 [72]	Cross-Sectional Study	Graduate and Undergraduate Students	93	Y

**Table 2:** Inclusion of measures of knowledge, attitudes, beliefs, and behavior.

Study	HPV				HPV Vaccine			
	Knowledge	Awareness	Attitudes/Beliefs	Behavior—HPV Screening	Knowledge	Awareness	Attitudes/Beliefs	Behavior—Vaccination Status
Afonso et al. 2017 [19]	Y	N	N	N	Y	Y	Y	Y
Albright et al. 2019 [20]	N	N	N	N	N	N	N	Y
Alsulami et al. 2023 [21]	Y	N	Y	N	Y	N	Y	Y
Barrera et al. 2021 [22]	N	N	N	N	N	N	Y	Y
Bennett et al. 2015 [23]	Y	N	Y	N	Y	N	Y	Y
Berenson et al. 2021 [24]	Y	N	Y	N	Y	Y	Y	Y
Berenson et al. 2017 [25]	Y	N	Y	N	Y	N	Y	Y
Berenson et al. 2020 [26]	Y	N	Y	N	Y	N	Y	Y
Blankenship et al. 2015 [27]	Y	N	Y	Y	N	N	N	N
Buchanan et al. 2015 [28]	N	N	N	N	Y	Y	Y	Y
Bunting et al. 2023 [29]	Y	N	N	N	Y	N	Y	N
Daniel et al. 2021 [30]	Y	N	Y	N	Y	N	Y	Y
D’Errico et al. 2020—JAANP [31]	N	N	N	N	Y	N	Y	Y
D’Errico et al. 2020 Journal of NP [32]	Y	N	Y	N	Y	N	Y	Y
Du et al. 2022 [33]	Y	N	N	N	Y	N	N	Y
Evans et al. 2020 [34]	Y	Y	N	N	N	N	Y	Y
Franca et al. 2023 [35]	Y	N	Y	N	Y	N	Y	Y
Frank et al. 2008 [18]	Y	N	N	N	N	N	N	N
Gerend and Magloire 2008 [36]	Y	N	Y	N	Y	N	Y	Y
Goldfarb et al. 2022 [37]	Y	Y	Y	N	Y	Y	Y	Y
Jacobson et al. 2020 [38]	Y	N	N	Y	N	N	N	N
Kepka et al. 2019 [39]	Y	N	Y	N	Y	N	Y	^33^N
Kim et al. 2019 [40]	Y	N	Y	N	Y	Y	Y	Y
Kubli et al. 2017 [41]	N	N	N	N	Y	N	Y	N
Laitman et al. 2018 [42]	Y	Y	N	N	N	N	N	N
Laitman and Genden 2018 [43]	Y	N	Y	Y	Y	N	Y	N
Laitman et al. 2020 [44]	Y	Y	Y	N	N	N	N	N
Levy et al. 2021 [45]	Y	Y	N	N	Y	Y	N	Y
Maginot et al. 2022 [46]	Y	Y	Y	N	Y	Y	Y	Y
McCready et al. 2015 [47]	Y	Y	Y	N	N	N	N	N
Moreno et al. 2017 [17]	N	N	N	N	Y	N	Y	N
Natipagon-Shah et al. 2021 [48]	Y	N	Y	N	Y	N	Y	Y
Navalpakam et al. 2016 [49]	Y	N	N	N	Y	N	Y	Y
Nesser and Ayodele 2023 [50]	Y	N	Y	N	N	N	N	Y
Nkwonta et al. 2022 [51]	Y	Y	Y	N	Y	Y	Y	Y
Patel et al. 2012 [52]	Y	N	Y	N	Y	N	Y	Y
Patel et al. 2013 [53]	N	N	Y	N	N	N	Y	N
Richman et al. 2016 [54]	Y	N	N	N	Y	N	N	Y
Richman et al. 2022 [55]	Y	Y	Y	N	Y	Y	Y	Y
Rohde et al. 2018 [56]	N	N	N	N	N	N	N	Y
Ruebsamen et al. 2018 [57]	Y	N	N	N	Y	N	N	N
Rutkoski et al. 2020 [58]	Y	N	N	N	Y	N	N	N
Schnaith et al. 2018 [59]	N	N	N	N	Y	Y	Y	Y
Shukla et al. 2022 [60]	Y	N	N	N	Y	N	Y	N
Suryadevara et al. 2016 [61]	Y	N	Y	N	Y	N	Y	Y
Sutton et al. 2023 [62]	Y	N	N	N	Y	N	Y	N
Thompson et al. 2017 [63]	N	N	N	Y	N	N	N	Y
Torres et al. 2022 [64]	Y	N	Y	N	N	N	Y	N
Tsau et al. 2011 [65]	N	N	Y	N	Y	N	N	N
Tung et al. 2019 [66]	Y	N	N	N	Y	N	Y	Y
Tung et al. 2021 [67]	N	N	N	N	Y	N	Y	Y
Walker et al. 2018 [68]	Y	Y	Y	N	Y	Y	Y	N
Wiley et al. 2018 [69]	Y	N	N	N	Y	N	N	N
Wiley et al. 2019 [70]	Y	N	N	N	Y	Y	Y	Y
Wright et al. 2021 [71]	Y	Y	Y	N	Y	Y	Y	N
Yacobi et al. 1999 [72]	Y	Y	N	N	Y	Y	N	N

## Appendix A. Complete Search String for Each Database Searched

Pubmed:
((((hpv) OR (human papillomavirus)) OR (hpv vaccin*)) OR (hpv immuni*)) AND (students [MeSH Terms])

CINAHL:
((((hpv) OR (human papillomavirus)) OR (hpv vaccin*)) OR (hpv immuni*)) AND ((MH students+))

Embase:
(‘hpv’ OR hpv OR ‘human papillomavirus’ OR ((‘human’/exp OR human) AND (‘papillomavirus’ OR papillomavirus)) OR ((‘hpv’ OR hpv) AND vaccin*) OR ((‘hpv’ OR hpv) AND immuni*)) AND student
